# Effects of WeChat use on the subjective health of older adults

**DOI:** 10.3389/fpsyg.2022.919889

**Published:** 2022-09-12

**Authors:** Ning Wei, Dingqiang Sun, Wenhao Huang

**Affiliations:** ^1^School of Management, Jiangsu University, Zhenjiang, China; ^2^College of Economics and Management, Nanjing Agricultural University, Nanjing, China; ^3^School of Management, Xuzhou Medical University, Xuzhou, China

**Keywords:** WeChat, social participation, social media, elderly, subjective health

## Abstract

**Objectives:**

In this study, the effect of WeChat use on the subjective health of older adults was examined.

**Methods:**

Using 2018 China Health and Retirement Longitudinal Study (CHARLS) survey data, we attempted to rule out potential endogeneity bias by employing instrumental variable regression to estimate the effects of WeChat use on the health of older individuals. Mobile phone price was chosen as the instrumental variable, and the health of older adults was measured from two aspects: self-rated health and the Center for Epidemiological Studies of Depression Scale (CES-D10).

**Results:**

WeChat use has a significant positive impact on the subjective health of older adults and can significantly improve their self-rated health and mental health status. WeChat use has significant positive effects on the subjective health of both older men and women, but relatively younger older adults obtain more health benefits from WeChat use.

**Conclusion:**

The virtual social participation prompted by WeChat use affects the health of older adults. This finding provides a reference for promoting the active integration of older adults into digital life.

## Introduction

With continuous advancements in living standards and medical technology, all countries have large aging populations. China is the world’s largest developing country and has an aging society with an ever-increasing aging population. According to the 2020 statistics of the National Bureau of China, as of the end of 2019, the population of older adults aged 60 years or older was approximately 254 million, accounting for 18.1% of the total population. Based on a forecast by the Ministry of Civil Affairs, at the end of the “Fourteenth Five-Year Plan,” China will become a “moderate aging” society, and the population of older adults aged 60 years or older will reach 300 million. China must address various factors that are inevitably brought about by an “aging society,” particularly the health of older adults. This problem is not present only in wealthy countries or regions; in fact, the burden of health issues in aging populations is more profound in low-and mid-income countries (WHO, 2015).

Social participation is an important way to achieve active aging and can play a positive role in promoting the health of older adults. In 2002, the World Health Organization (WHO) proposed the concept of “active aging,” which has become the main reference frame of the WHO, the Organization for Economic Cooperation and Development (OECD) and other international organizations in formulating health policies for older adults. The goal of active aging is to improve the quality of life of every older individual so that these individuals can attain optimal health, social participation, and safety. As one component of active aging, social participation is a constructive activity in which individuals and groups voluntarily or actively participate in community activities, allowing participants to benefit from interacting and communicating with each other (Tomaka et al., 2006). For older adults, social participation can be identified as the promotion of affairs of common interests through enhanced interpersonal interactions between an individual and his/her relatives and friends or community in a social network or organized group (Dorfman, 1995). According to theories related to social participation, older adults have different levels of social participation due to different levels of family social support (Lian et al., 1999). The higher the level of social participation by older adults is, the better their interpersonal relations, the higher their life satisfaction and the better their health. Social participation is an important support system to maintain the interpersonal relations of older adults; such relations include participation in educational, political, organizational, religious, volunteer and other social activities (Kuo et al., 2004). Social participation by older adults can include formal activities, such as volunteering and community activities, as well as informal activities, such as telephone conversations and social interactions with friends (Utz et al., 2002).

With the continuous development of China’s internet industry, the penetration rate of smartphones and tablet computers among older adults has been continuously increasing. According to the 47th Report of China’s Internet Development Status by the Chinese Internet Network Information Center in 2021, as of December 2020, 986 million Chinese people accessed the internet through mobile phones, 99.7% of whom solely used mobile phones to access the internet; the population aged 60 years or older accounted for 11.2% of the total users. It is estimated that approximately 110 million older adults access the internet through mobile phones, accounting for 43.7% of the older population. The increase in smartphone use by the older population has also brought about changes in the way older adults participate in social activities, such as the use of social media. WeChat is China’s largest social media platform. According to data from the Tencent Research Institute, the numbers of active WeChat users aged 55 years or older were 7.68, 50, and 61 million in 2016, 2017, and 2018, respectively. Additionally, WeChat use by older adults is specific, with 85% using WeChat for social functions. The ability and scope of social participation of older adults declines with age; however, social participation directly affects the health status of older individuals. If traditional social participation can promote the health of older adults, it is necessary to determine whether the use of WeChat has an impact on their health. Considering the very large number of older adults in China, an increasing number of older adults have begun to use social media to achieve social participation, and the close relationship between WeChat use and the health of older adults deserves more attention. The investigation of this issue is of great theoretical and practical significance for society and for actively coping with the aging population, especially for improving the health of older adults in the digital age.

Unlike previous studies on the effect of social media use on users’ health, we expanded our study in the following aspects. First, we incorporated an investigation into the effect of social participation on the health of older adults and included virtual social participation through WeChat as a factor that affects the health of older adults. Second, because social media was first popular among young people and older adults have demonstrated low acceptance of the internet and smartphones, previous studies on this topic have all focused on young people. With the continuous penetration of social media in the population, increasingly more older adults have begun to use social media. However, the effect of social media use on the health of older adults has not been examined and is thus addressed in this study for the first time. Third, considering that social media use and the health of older adults interact, in this study, we innovatively used mobile phone use by older adults as an instrumental variable to eliminate the possible endogeneity problem of ordinary least squares (OLS) estimation. Finally, there are obvious gender differences in the relationship between social media use and health, especially among young people, but whether they are also present among older adults is unknown; therefore, this issue was examined in this study.

Based on the above discussion and the 2018 survey data from the China Health and Retirement Longitudinal Study (CHARLS), we used older adults in China as the subjects in this study to investigate the effect of WeChat use on their subjective health in the context of increasing numbers of Chinese older adults using WeChat. The endogeneity problem was solved using the instrumental variable method. We attempted to answer the following questions: how does WeChat use affect the subjective health of older adults? How does this effect vary?

## Related literature

The content of social participation activities is quite expansive, including activities with respect to religion, volunteering, leisure, and health (Park et al., 2014). For older adults, social participation may be a single activity (Sirven and Debrand, 2012) or the total time an individual spends on a series of social events (Baker et al., 2005). With advances in technology, social media, referring to various internet-based network platforms that enable users to interact verbally and visually with others (Carr and Hayes, 2015), is considered by older adults to be a means of strengthening social networks (Silva et al., 2018) and promoting social participation and intergenerational communication in addition to the traditional social participation content (Nef et al., 2013). As exchanges on social media have gradually become an increasingly popular form of social interaction, social networking sites (SNSs), such as Facebook, Instagram and Twitter, have also become a ubiquitous part of everyday life (Vahedi and Zannella, 2019). The emergence of social media has allowed people to use virtual networks for social communications; therefore, they are not limited to face-to-face social networks in the real world. The impact of social media on health caused by this change has also begun to attract scholars’ attention.

The use of social media has a positive impact on many aspects of health, including reducing anxiety and loneliness, alleviating depression, increasing happiness, and promoting physical health (Gilmour et al., 2020). Social media can increase social support, strengthen an individual’s association with the real world and allow people to fully express themselves and obtain social support (Ballantine et al., 2015; Procentese et al., 2019). According to a study by Grieve et al. (2013), the higher the level of social connection through Facebook is, the lower the levels of depression and anxiety and the higher subjective well-being (life satisfaction) is Seo et al. (2016) investigated the possible impact of Facebook-based social support on loneliness and found that more social interactions with Facebook friends and faster responses to posts from these friends increase Facebook users’ perceived social support and ultimately reduce loneliness. Self-expression on social media has a positive effect on easing loneliness, enhancing the perceived social support of social media users and thus improving an individual’s well-being (Lee et al., 2013). Moreover, the more Facebook-based social support an individual receives, the higher his or her level of happiness (Hu et al., 2017; Kohout and Schumann, 2020). Nabi et al. (2013) showed that the number of friends on Facebook is correlated with a higher level of perceived social support, which in turn can reduce psychological pressure, thereby improving the user’s physical health and happiness. Although this effect is minimized when considering the size of interpersonal networks, for individuals who have experienced many objective life stresses, the number of Facebook friends is a strong predictor of perceived social support. Cavallo et al. (2014) analyzed the relationship between social support and sports engagement in social networks and found that an increase in support from peers is positively correlated with an increase in physical exercise intention and an indirect increase in an individual’s physical activity. In addition, an increase in support received through Facebook (e.g., encouragement related to athletic ability) is also positively correlated with an increase in physical activity.

However, other studies have shown that social media use can reduce face-to-face interactions between people as well as users’ participation in meaningful social activities and that sedentary behavior leads to increased screen time (Guernsey, 2014). Sedentary behavior caused by social media use greatly increases the risk of physical health problems (Silva et al., 2017). The use of social media may also lead to internet addiction. Although internet addiction and a reduction in meaningful social activities may be related to any internet activity, unfavorable social comparisons are often highly correlated with the use of social media (Vogel et al., 2015) and harm individual self-esteem, thereby increasing the risk of mental health problems (Feinstein et al., 2013). People tend to show the best side of their lives on social media, leading others to compare their own living conditions with those that their friends show on social media, which generates a negative impact, damages self-esteem and, in turn, leads to depression (Pera, 2018; Tosun and Kadarma, 2019). The negative effects of Facebook use on mental health include increased anxiety and depression, excessive attention to body image, disordered eating, and alcoholism (Gilmour et al., 2020). However, past research has also shown that there is no correlation between the use of social media and health (Jelenchick et al., 2013).

*Hypothesis 1*: WeChat use has a significant positive impact on the subjective health of older adults.

The gender difference regarding the impact of social media on health has been investigated previously. Neira and Barber (2014) showed that social media use may have a negative impact on the health of young women but a positive impact on the health of young men. Frison and Eggermont (2016) found that active disclosure on Facebook increases boys’ depressive emotions and that passive use of Facebook increases girls’ depressive emotions. Additionally, perceived online social support significantly regulates the relationship between active Facebook use and girls’ depressive emotions, and active Facebook use increases girls’ perceived online social support, which in turn reduces girls’ depressive emotions. Kelly et al. (2018) evaluated the potential impact of prolonged use of social media on the mental health of youth and found that the association between social media use and depression symptoms is higher among girls than among boys.

Tsitsika et al. (2014) showed that age has a significant effect on the relationship between heavy use of social media and negative internalization; compared with older social media users, younger social media users are more likely to experience negative internalization (e.g., fear, physical complaints, worry, shyness, and other psychological abnormalities).

Chan (2018) investigated the relationship between smartphone use (voice, email, SMS, Facebook, and WhatsApp) and subjective well-being and found that in people of all ages, face-to-face communication, and satisfaction with friendships are correlated with mental health and positive emotions, and mobile communication is correlated with satisfaction with friendships and social support among older people (aged 35–54 years and 55–70 years or above). Facebook use and the number of Facebook friends are correlated with the mental health and perceived social support of individuals aged 18–34, while WhatsApp use is correlated with perceived social support in all age groups.

*Hypothesis 2*: There is significant heterogeneity in the impact of WeChat use on the subjective health of older adults.

In summary, social participation contributes to improvements in the health status of older adults. However, the effect of social media, as a new form of social participation, on the health of older adults remains controversial. In addition, because the use of social media, especially social applications, is becoming more dependent on smartphones, previous studies on this topic have focused on youth, a group with high smartphone use, while the older population has rarely been examined. However, as the popularity of smartphones increases among older adults, it is necessary to analyze the impact of social media on the health of this group. Therefore, in this study, we chose the older population in China as subjects to examine the impact of WeChat use on their subjective health.

## Materials and methods

### Data source

The data used in this study were obtained from CHARLS, which was sponsored by the National School of Development of Peking University and implemented by the Institution of Social Science Surveys of Peking University in 2018. Starting in 2011, nationwide surveys were conducted through CHARLS to study China’s aging population and promote interdisciplinary research on aging. The sample obtained from CHARLS included 18,000 random individuals aged 45 or above from more than 10,000 households in 450 villages, 150 counties, or districts of 28 provinces (autonomous regions or municipalities directly under the central government) in China. The survey in 2018 was the 4th routine follow-up survey of national baseline samples and included a total of 8,198 samples, which were further screened in this study. A total of 5,442 valid samples were retained after excluding those with missing data for the study variables.

### Variable selection

The measurement of subjective health in this study included two aspects: self-rated health and mental health. Data for self-rated health were obtained directly from the CHARLS questionnaire, for which respondents used a 5-point Likert scale (1 = very good; 2 = good; 3 = fair; 4 = bad; 5 = very bad); the lower the score, the better the self-rated health status. Data for mental health were obtained using variables of depression. In the “health and function” module of the CHARLS questionnaire, respondents complete the CES-D10, a short version of the Center for Epidemiological Studies Depression scale, which contains two items on positive emotions, five items on physical symptoms and three items on depressive emotions that are answered with “rarely or not at all (<1 day),” “not frequently (1–2 days),” “sometimes or half of the time (3–4 days),” or “most of the time (5–7 days).” In reference to the methods of previous studies, we reversed the order of the respondent’s choice when answering the positive emotion items so that the order of all items consistently reflected the respondent’s degree of depression, which was given a value of 0, 1, 2, or 3. Therefore, the sum of the scores of the 10 items (range of 0–30 points) reflected the respondent’s degree of depression; the higher the score, the higher the degree of depression (Zimmerman and Katon, 2005).

To assess WeChat use by older adults, the following question was used in the CHARLS questionnaire: “Do you use WeChat?” (1 = use, 0 = not use). Personal and household variables were used as control variables. Personal variables included age, gender, education, income, marital status, and objective physical status (number of chronic diseases and activities of daily living). Household variables included support of children and place of residence. Table 1 provides the definition of each of the abovementioned variables and the descriptive statistics.

**Table 1:** Variable definitions and descriptive statistics (China Health and Retirement Longitudinal Study, China, 2018).

Variable name	Definition	Mean	Standard deviation	Minimum value	Maximum value
Self-rated health	1 = very good, 2 = good, 3 = fair, 4 = bad, 5 = very bad	3.12	1.04	1	5
Mental health	Measured with the self-rating CES-D10	8.73	6.68	0	30
WeChat use	1 = yes, 0 = no	0.05	0.23	0	1
Phone price	Continuous variable (yuan)	501.97	1053.54	0	30,000
Age	Continuous variable	69.34	7.23	60	92
Gender	1 = male, 2 = female	1.51	0.50	1	2
Marital status	1 = married, 0 = single	0.75	0.44	0	1
Education level	Continuous variable	3.00	1.91	1	11
Household income	Logarithm of annual income	9.64	1.46	3	17
Residential area	1 = urban, 0 = rural	0.27	0.44	0	1
Residential arrangement	1 = live with children, 0 = live independently	0.52	0.50	0	1
Chronic disease	Number of chronic diseases	0.80	1.11	0	11
Disability	Number of activities of daily living unable to perform	0.28	0.94	0	6

Regarding the education variable, in reference to the question in the CHARLS questionnaire, different values are assigned for various answers: “no education (illiterate) = 1,” “did not finish elementary school = 2,” “finished old-style private school = 3,” “finished elementary school = 4,” “finished junior high school = 5,” “graduated from high school = 6,” “graduated from technical secondary school (including secondary normal school and secondary specialized school) = 7,” “graduated from vocational college = 8,” “graduated from college = 9,” “earned a master’s degree = 10” and “earned a PhD = 11.”

### Model setting

To test the hypotheses proposed in this study, we used a multivariate linear regression model to estimate the impact of WeChat use on the subjective health of older adults:


(1) $$Y_{i}=\alpha +\beta APP_{i}+\theta X_{i}+\varepsilon_{i}$$

where $Y_{i}$ is subjective health status, including self-rated health, and mental health; $APP_{i}$ is WeChat use; $X_{i}$ is the control variables, including the personal variables (e.g., age, gender, education, income, marital status, and physical condition) and household variables (e.g., living with children or not, place of residence); and $\varepsilon_{i}$ is a random disturbance item.

### Endogeneity analysis

Endogeneity refers to the estimation of regression equations in which one or more explanatory variables in the model are correlated with random disturbance terms, resulting in biased parameter estimates (Cameron and Trivedi, 2005). Due to the significant difference between the health status of older adults and their degree of social participation, older adults with good health are more willing to engage in social participation than those with poor health (Kuo et al., 2004). Therefore, when used as an explanatory variable, social participation may cause endogeneity, leading to important information being overlooked when analyzing causality and thus to overestimation of the positive features of social participation (Dawson-Townsend, 2019). Therefore, WeChat use in model (1) may have generated endogeneity due to missing variables or reverse causality. When an older person has certain health problems, he/she may start to use WeChat to increase social participation and seek social support; therefore, reverse causality between WeChat use and health should not be ignored. In addition, whether an older person uses WeChat may be affected by other factors, such as personal habits, the surrounding environment, and the ability to accept new things, which are unobservable.

In this study, we adopted an instrumental variable method to address endogeneity and chose mobile phone price as the instrumental variable; WeChat use is possible only if one owns a smartphone, and WeChat use by older adults is correlated with the price they paid for their smartphone without having a direct impact on their health. Therefore, this instrumental variable is theoretically feasible.

### Robustness test

As discussed above, an instrumental variable was used to address the endogeneity problem. To ensure robust empirical results, we further adopted propensity score matching (PSM) to address endogeneity. PSM constructs an “anti-fact” frame that approximates a “randomized trial” to avoid the error due to confounding factors that leads to the so-called “net effect” when comparing effects between a treated group and control group. We were interested in the net effect of WeChat use on the subjective health of older adults, i.e., the average treatment effect on the treated (ATT). ATT can be estimated using the following formulas:


(2) $$Y_{i}=Y_{0i}+\left(Y_{1i}-Y_{0i}\right)D_{i}$$

(3) $$ATT=E\left(Y_{1i}-Y_{0i}|D_{i}=1\right)$$

In Formula (2), $D_{i}$ is the treatment variable. When $D_{i}$ = 1, individual *i* is the treated group, i.e., an older person who uses WeChat, and when $D_{i}$ = 0, individual *i* is the control group, i.e., an older person who does not use WeChat. Formula (3) estimates the ATT of the treated group, i.e., the net effect of WeChat on the subjective health of older adults.

## Results

### Regression results

Table 2 shows the estimates of WeChat’s impact on the self-rated health and mental health of older adults. Because the dependent variable in Formula (1) is the subjective health status of older adults, which includes self-rated health and mental health (i.e., two continuous variables), we adopted an OLS model to estimate the dependent variable. Models 1 and 3 are the estimation results obtained through the OLS model, while Models 2 and 4 are the estimation results obtained through the two-stage least-square (2SLS) model. Among the 2SLS estimation tests, the chi-square of the C statistic of the endogeneity test was 8.09, which is statistically significant at the significance level of 1%, indicating that the WeChat use variable introduced endogeneity. The weak identification test results for the instrumental variable model showed that the *F* value of the Cragg-Donald Wald test was 76.87, which is much greater than 10, indicating that the selected instrumental variable in the model is not weak. Models 2 and 4 also generated regression results for the first stage of the 2SLS model, indicating that mobile phone price has a significant impact on WeChat use by older adults and that the variable is statistically significant at the 1% level.

**Table 2:** Effects of WeChat use on the self-rated health and mental health of older adults [ordinary least squares (OLS), two-stage least squares; China Health and Retirement Longitudinal Study, China, 2018].

Variable name	Model 1	Model 2		Model 3	Model 4		Stage 1	Stage 2	Stage 1	Stage 2
WeChat use	−0.213^***^		−1.157^***^	−1.600^***^		−6.777^***^
	(−3.932)		(−3.289)	(−4.185)		(−2.949)
Phone price		0.001^***^			0.001^***^	
		(13.300)			(13.300)	
Age	0.001	−0.002^***^	0.001	0.074^***^	−0.002^***^	0.085^***^
	(−0.064)	(−5.124)	(−0.732)	(−5.513)	(−5.124)	(−5.198)
Gender	0.015	0.013^**^	0.049^*^	1.284^***^	0.013^**^	1.368^***^
	(0.640)	(2.403)	(1.896)	(7.294)	(2.403)	(7.069)
Education	0.001	0.021^***^	0.023^**^	−0.343^***^	0.021^***^	−0.228^***^
	(0.118)	(13.528)	(2.153)	(−6.687)	(13.528)	(−2.800)
Marital status	0.005	0.001	−0.008	−0.991^***^	0.001	−1.130^***^
	(0.192)	(0.083)	(−0.275)	(−4.848)	(0.083)	(−4.939)
Household income	−0.054^***^	0.009^***^	−0.052^***^	−0.557^***^	0.009^***^	−0.469^***^
	(−6.591)	(4.579)	(−5.074)	(−8.397)	(4.579)	(−5.767)
Residential area	−0.074^***^	0.058^***^	−0.006	−0.994^***^	0.058^***^	−0.602^**^
	(−2.640)	(8.878)	(−0.153)	(−4.656)	(8.878)	(−2.178)
Residential arrangements	0.040*	−0.019^***^	0.022	0.209	−0.019^***^	0.139
	(1.867)	(−3.737)	(0.914)	(1.268)	(−3.737)	(0.756)
Chronic disease	0.172^***^	0.004^**^	0.171^***^	0.839^***^	0.004^**^	0.851^***^
	(18.667)	(1.996)	(16.618)	(11.560)	(1.996)	(10.834)
Disability	0.216^***^	−0.009^**^	0.238^***^	1.821^***^	−0.009^**^	1.848^***^
	(17.312)	(−2.535)	(14.799)	(12.444)	(−2.535)	(10.937)
Constant term	3.590^***^	−0.004	3.603^***^	19.362^***^	−0.004	19.057^***^
	(23.541)	(−0.115)	(20.562)	(15.535)	(−0.115)	(13.863)
Number of samples	5,442	5,442	5,442	5,442	5,442	5,442
Endogeneity test			8.09^***^			8.09^***^
Weak instrumental variable test			76.87^***^			76.87^***^

(1) The endogeneity test generates the *C* statistic (chi^2^ value), and the weak instrumental variable test generates the Cragg-Donald Wald statistic (*F*-value); (2) The value in parentheses is the standard error of heteroscedasticity; (3) ^*^, ^**^, and ^***^ represent statistical significance at the 10, 5, and 1% levels, respectively.

First, as shown in Table 2, in Models 1 and 2, the estimated coefficient of the WeChat use variable was significantly negative at the 1% level, indicating that the self-rated health of older adults who use WeChat is significantly higher than that of older adults who do not use WeChat. Therefore, WeChat use can improve the SRH of older adults to a certain extent. Second, as shown in Table 2, from the perspective of the impact of WeChat use on the mental health of older adults, the estimated coefficient of the WeChat use variable was statistically significant at the 1% level in Models 3 and 4. This finding indicates that WeChat use also has a significant impact on the mental health of older adults. Therefore, WeChat use by older adults significantly enhances their subjective health.

In the four models, the results for the control variables all indicated that the self-rated health and mental health of older adults decreased significantly with age, the subjective health status of men was better than that of women, and a higher education level increased the subjective health status of older adults. The type of household registration also had a significant impact on the subjective health status of older individuals; older adults in urban areas had higher subjective health status than those in rural areas. The impact of objective physical condition variables on the health status of older adults was also statistically significant, i.e., the poorer the objective physical condition of older adults, the poorer their self-rated health and mental health.

### Robustness analysis

#### PSM estimation

Sample selection bias can affect the reliability of the regression results. Whether an older person uses or does not use WeChat is not random and may be affected by other factors. Therefore, a direct comparison of the subjective health differences between the two groups of older adults, i.e., “older adults who use WeChat” (treated group) and “older adults who do not use WeChat” (control group), would lead to biased results. The PSM method was used because it can not only solve the endogeneity problem but also calculate the ATTs for the treated and control groups and indicate robustness. To ensure robust estimation results, we adopted three matching methods: nearest neighbor matching, radius matching (with a radius of 0.01) and kernel matching (with the default kernel function and bandwidth).

In accordance with the analysis steps of the PSM method, we first estimated the probability of an older respondent being placed in the treated group (older adults who use WeChat). The covariates in the probability model included the personal characteristics of older respondents (e.g., age, gender, education, income, marital status, and objective physical condition) and household characteristics (e.g., children’s support, place of residence). Then, matching was conducted based on the probability, and the differences in self-rated health and mental health with the matched sample group (i.e., the ATTs of the treated group) were calculated.

Table 3 provides the ATT results calculated using the three matching methods. First, the self-rated health of older adults who used WeChat was significantly higher than that of older adults who did not use WeChat. The difference between the two groups depended on the matching method used but was highly significant, which verifies the conclusions in Table 2 to a certain extent, indicating that the conclusions of this study are robust. Second, older adults who used WeChat and those who did not use WeChat differed significantly in average mental health, indicating that WeChat use significantly affects the mental health of older adults, which is consistent with the conclusions in Table 2 and further shows that the results of this study are relatively robust.

**Table 3:** Propensity score matching results (propensity score matching; China Health and Retirement Longitudinal Study, China, 2018).

	Average treatment effect	Standard error	*T*-value
**Dependent variable: self-rated health**			
Nearest neighbor matching	−0.187^**^	0.075	−2.50
Radius matching	−0.347^***^	0.045	−7.63
Kernel matching	−0.203^**^	0.075	−2.48
**Dependent variable: mental health**			
Nearest neighbor matching	−1.326^***^	0.487	−2.72
Radius matching	−3.645^***^	0.291	−9.60
Kernel matching	−1.676^***^	0.360	−4.66

^*^, ^**^, and ^***^ represent statistical significance at the 10, 5, and 1% levels, respectively.

Eliminating samples that might have not actually used WeChat.

The data used in this study were collected through the question “Do you use WeChat?” in the CHARLS survey. Taking into account actual WeChat use, there was another question in the CHARLS survey, i.e., “Do you use WeChat Moments?,” which allowed us to limit the older population who use WeChat to those who use WeChat and WeChat Moments. Table 4 provides the regression results after excluding the samples that might have not actually used WeChat. For the OLS and the 2SLS estimation methods, the regression results are consistent with those shown in Table 2, indicating that WeChat use has a significant impact on the self-rated health and mental health of older adults and that the impact is slightly more profound than the results shown in Table 2.

**Table 4:** Effects of WeChat use on the subjective health of older adults (only those who use WeChat Moments; OLS, two-stage least squares; China Health and Retirement Longitudinal Study, China, 2018).

Variable name	Self-rated health		Mental health	
	Model 5	Model 6	Model 7	Model 8
WeChat use	−0.222^***^	−1.200^***^	−1.500^***^	−7.142^***^
	(−3.589)	(−3.318)	(−3.428)	(−3.030)
Control variable	Controlled	Controlled	Controlled	Controlled
Constant term	3.587^***^	3.602^***^	19.267^***^	18.973^***^
	(23.373)	(20.477)	(15.324)	(13.685)
Sample size	5,356	5,356	5,356	5,356
Endogeneity test		8.10^***^		8.10^***^
Weak instrumental variable test		197.10^***^		97.10^***^

(1) The endogeneity test generates the *C* statistic (chi^2^ value), and the weak instrumental variable test generates the Cragg-Donald Wald statistic (*F*-value); (2) The value in parentheses is the standard error of heteroscedasticity; (3) ^*^, ^**^, and ^***^ represent statistical significance at the 10, 5, and 1% levels, respectively.

### Analysis of heterogeneity

#### Gender difference

Table 5 shows the gender-based estimation results for the impact of WeChat use on the subjective health of older adults. When self-rated health was used as the dependent variable, the coefficient of the WeChat use variable was significantly negative, indicating that in the older population, the self-rated health of both men and women is enhanced by WeChat use and that men are affected more profoundly. Similarly, in terms of mental health, the use of WeChat had a significant impact on men and women, and in terms of the impact coefficient, WeChat use reduced the depressive emotions of older women more significantly than those of older men.

**Table 5:** Subsample test results (two-stage least squares; China Health and Retirement Longitudinal Study, China, 2018).

Variable name	Grouping by gender			
	Self-rated health		Mental health	
	Men	Women	Men	Women
WeChat use	−1.450^***^	−0.966^**^	−6.900^**^	−7.124^**^
	(−2.770)	(−2.003)	(−2.176)	(−2.153)
Control variable	Controlled	Controlled	Controlled	Controlled
Constant term	3.652^***^	3.661^***^	19.218^***^	23.248^***^
	(14.817)	(15.961)	(11.060)	(11.730)
Sample size	2,542	2,900	2,542	2,900
	**Grouping by age**			
	**Self-rated health**		**Mental health**	
	**Younger than 70 years**	**70 years or older**	**Younger than 70 years**	**70 years or older**
WeChat use	−1.568^***^	−0.187	−9.345^**^	−5.781*
	(−3.421)	(−0.286)	(−2.109)	(−1.913)
Control variable	Controlled	Controlled	Controlled	Controlled
Constant term	3.903^***^	3.554^***^	21.460^***^	19.017^***^
	(9.882)	(10.249)	(10.846)	(6.257)
Sample size	3,551	1891	3,551	1,891

(1) The value in parentheses is the standard error of heteroscedasticity; (2) ^*^, ^**^, and ^***^ represent statistical significance at the 10, 5, and 1% levels, respectively.

#### Age difference

Table 5 also shows the regression results for age groups. WeChat use had a significant impact on the self-rated health of older adults younger than 70 years but not on that of older adults aged 70 years or older, whereas it had a significant impact on the mental health of older adults in all age groups. The absolute value of the regression coefficient increased with increasing age group, i.e., the effect of WeChat use on the subjective health of older adults differed significantly between different age groups. As older adults age, WeChat use has less of a positive impact on the subjective health of older adults.

## Discussion

This paper estimates the impact of WeChat use on the health of older adults while addressing endogeneity problems. Using CHARLS 2018 data, the empirical test found a significant positive impact of WeChat use on the health of older adults through an instrumental variables model. This finding is consistent with the findings of existing studies on samples of adolescents (Grieve et al., 2013; Gilmour et al., 2020). The impact of social engagement on the health of older adults gives this study a starting point for further research (Ang, 2018). Traditional studies on the impact of social engagement on older adults’ health have many shortcomings, which are insufficient in supporting theory development and innovation research. In terms of research content, traditional social engagement has been gradually replaced by an increasing number of virtual social engagements on social media, and a large number of older adults have started to use social media for social engagement. At the methodological level, existing studies have not considered the endogeneity issue. According to the literature review, social engagement, as an explanatory variable, may suffer from endogeneity issues, which can lead to an overemphasis on positive functions while omitting other important information in causality analysis (Dawson-Townsend, 2019). Therefore, endogeneity issues must be taken into account when estimating the health effects of social media use. In this study, the value of cell phones owned by older adults was used as an instrumental variable to derive unbiased estimates through the use of an instrumental variable model. The potential influence of heterogeneous factors was also controlled as much as possible through PSM estimation, and robustness tests were performed on the empirical results to confirm the existence of this self-selection bias. Building on the empirical examination of the impact of WeChat use on the health of older adults, this paper further explores the heterogeneity of this impact.

Combined with the results of existing studies focusing on adolescent populations, studies have found gender differences (Neira and Barber, 2014) as well as age differences (Tsitsika et al., 2014) in the health effects of social media use. This study also found gender differences and age differences. In particular, this study found that WeChat use has significantly different levels of subjective health effects on male and female older adults, with women being more affected in terms of mental health. Our findings are similar to those revealed by studies with younger adults (Kelly et al., 2018). One possible explanation is that when female older adults use WeChat, they are better at sharing their life and psychological status with family and friends, thus forming active social interactions, while it is more difficult for male older adults to do so; for them, WeChat use is more a channel for information transmission than a tool for social interaction.

Relatively younger older adults were able to derive more health benefits from WeChat use, which is similar to the findings of a study conducted on a younger age group (Chan, 2018). One possible reason for this phenomenon is that older adults still face barriers to using WeChat, especially in the older age group. The younger cohort of older adults is better able to learn and accept new things, and thus they are more likely to integrate into the digital life that comes with WeChat use and to experience greater mental health benefits. Although the oldest adults in the sample also used WeChat, they received fewer mental health benefits from WeChat use because they lacked a comprehensive grasp of its features and thus were unable to fully use it.

## Conclusion and policy implications

In the context of the increasing use of WeChat by Chinese older adults, we examined the effect of WeChat use on the subjective health of older adults in China using 2018 CHARLS data. We found that WeChat use has a significant positive impact on the subjective health of older adults and can significantly improve the self-rated health and mental health of older adults. Considering that there may be heterogeneity in the impact of WeChat use on the health of older adults, we analyzed heterogeneity from two aspects: gender and age. We found that WeChat use has a significant positive impact on the subjective health of both older men and older women but that older men are affected more profoundly in terms of self-rated health, while older women are affected more profoundly in terms of mental health. In addition, relatively younger older adults obtain more health benefits from WeChat use.

In the context of China’s increasingly aging population, with the development of the Internet and mobile phones, the use of WeChat has gradually become a major form of social participation for older adults in China, providing a new and convenient way for them to enhance communication and exchanges. As increasingly more older adults use WeChat as a mode of online social participation, the close link between the health of older adults and their WeChat use requires an in-depth examination. In this study, we show that WeChat use can enhance the self-rated health of older adults and reduce their level of depression. WeChat, as an important form of social media, has gradually played an increasingly important role in the social participation of older individuals. Therefore, at the family level, it is necessary to address the use of WeChat by older adults because it will provide a new way to facilitate family interactions in which children need to assume the responsibility of teaching older adults how to use WeChat and share a digital life with them. At the social level, it is necessary to create a friendlier internet environment for older adults to help them gradually integrate into the rapidly developing networking era and explore ways to improve their digital life so that they can enjoy technology as much as the young generation. Moreover, it is necessary to address the actual needs of older adults when engaging in social participation and the role WeChat plays in enabling new digital social communications. Doing so requires the joint efforts of the family and society as a whole as well as the support of relevant functional departments of the state.

This study has some limitations. First, some variables might have been overlooked. We did not control for environmental factors that influence the use of WeChat by older adults because questions about mobile phone use in CHARLS are limited, making it impossible to understand the environmental conditions of WeChat use by older adults. For example, we could not examine WeChat use by friends with whom older adults share moments and whether older adults received assistance and support from family members when using WeChat. These environmental factors can have an impact on whether an older person uses WeChat; however, this information is missing in the CHARLS data, making it impossible to examine the effect of these factors on WeChat use by older adults. Second, in this study, we examined the effect of WeChat use on the subjective health of older adults. However, the details of WeChat use by older adults, including active or passive WeChat use, number of WeChat friends, WeChat use time, WeChat addiction, etc., should also be included. Unfortunately, due to limitations related to the questionnaire survey data, it was not possible to conduct more detailed analyses.

## Data availability statement

The original contributions presented in the study are included in the article/supplementary material, further inquiries can be directed to the corresponding author.

## Author contributions

All authors were involved in planning of the study and interpretation of results. NW and WH conducted the data analysis and wrote the first draft of the paper. DS critically reviewed the paper and edited the manuscript. All authors contributed to the article and approved the submitted version.

## Funding

We would like to acknowledge the support of the Ministry of Education in China (MOE) Project of Humanities and Social Sciences (Project no. 17YJC840041).

## Conflict of interest

The authors declare that the research was conducted in the absence of any commercial or financial relationships that could be construed as a potential conflict of interest.

## Publisher’s note

All claims expressed in this article are solely those of the authors and do not necessarily represent those of their affiliated organizations, or those of the publisher, the editors and the reviewers. Any product that may be evaluated in this article, or claim that may be made by its manufacturer, is not guaranteed or endorsed by the publisher.
